# Enhancing health literacy and patient engagement through a privacy-aware social network-based digital health communication framework

**DOI:** 10.3389/fpubh.2026.1908384

**Published:** 2026-09-15

**Authors:** Nasser Allheeib, Salman Khan, Farah Mohammad, Dost Muhammad Khan

**Affiliations:** 1 Department of Information Systems, College of Computer and Information Sciences, King Saud University, Riyadh, Saudi Arabia; 2 Department of Computer Science, College of Computer and Information Sciences, King Saud University, Riyadh, Saudi Arabia; 3 Department of Computer Science, University College of Duba, University of Tabuk, Tabuk, Saudi Arabia; 4 School of Mathematics and Statistics, University of Glasgow, Glasgow, United Kingdom

**Keywords:** digital health communication, health communication systems, health education and promotion, health literacy, patient engagement, privacy in digital health, social health networks

## Abstract

Digital health applications and social-network-enabled healthcare platforms have transformed healthcare delivery by facilitating health communication, patient engagement, and access to medical information. However, the continuous exchange of sensitive biomedical data across interconnected digital environments raises significant ethical, privacy, and security challenges that may compromise user trust and the effective adoption of digital health technologies. Existing studies primarily address individual security and privacy concerns, with limited attention to integrated frameworks that simultaneously support ethical data governance, secure communication, and digital health education. This study proposes a privacy-preserving digital health communication framework that integrates social-network-based healthcare applications with a multi-cloud infrastructure, distributing healthcare data and services across multiple independent cloud service providers to support system availability, reliability, fault tolerance, and secure information management. The proposed methodology combines a healthcare communication architecture, role-based access control, and stakeholder specific privacy policies to facilitate secure information exchange among patients, healthcare professionals, caregivers, and emergency responders while supporting the ethical handling of biomedical data. The framework is conceptually evaluated by analyzing healthcare communication workflows, user roles, privacy requirements, and security threats across representative healthcare communication scenarios, including chronic disease monitoring, emergency response, and post-discharge care coordination. This analysis suggests that the proposed framework may support secure information sharing, improve coordination among healthcare stakeholders, strengthen patient awareness and health literacy, and facilitate timely healthcare decision-making while preserving data confidentiality and user privacy. By integrating privacy-preserving mechanisms with digital health communication, the proposed framework is designed to support secure digital health education and public health promotion, contributing conceptually to Sustainable Development Goals 3 (Good Health and Well-being), 4 (Quality Education), and 17 (Partnerships for the Goals).

## 1 Introduction

The rapid advancement of mobile health (mHealth) technologies, social-network-based applications, and digital healthcare platforms has fundamentally transformed the delivery, communication, and management of healthcare information. These technologies have become integral components of modern healthcare by facilitating patient-provider communication, supporting health education, promoting patient engagement, and improving access to healthcare services in both clinical and community settings. Social networking platforms have also emerged as valuable environments for disseminating health information, encouraging peer support, and strengthening collaboration among patients, caregivers, and healthcare professionals (1, 2). Furthermore, social network analysis has been widely employed to investigate communication patterns, knowledge dissemination (3), and relationships among individuals and healthcare organizations, providing valuable insights into healthcare collaboration and information exchange (4). The growing adoption of digital health platforms offers numerous benefits, including improved access to reliable health information, enhanced patient engagement, support for disease self-management, and increased opportunities for health education and promotion (5, 6). Through online communities and digital communication platforms, patients can actively participate in healthcare discussions, share experiences, and receive continuous support from healthcare professionals and peers, thereby strengthening health literacy and informed decision-making. Recent advances in wearable devices and mHealth applications have further expanded digital healthcare by enabling continuous patient monitoring, remote healthcare delivery, and real-time exchange of biomedical data (7, 8).

Despite these advantages, privacy, security, and the ethical management of sensitive biomedical data remain major barriers to the widespread adoption of digital health technologies. The continuous collection, storage, and exchange of sensitive biomedical data increase the risks of privacy breaches, unauthorized access, and information leakage, as well as ethical concerns about data governance. Previous studies have reported concerns including data leakage, unauthorized access, limited user trust, and insufficient regulatory frameworks governing digital health environments (9, 10). Users’ willingness to adopt online healthcare systems is strongly influenced by their trust in the platform, perceived usefulness of the services, and confidence in the reliability of shared health information (11). Integrating interconnected healthcare technologies into existing healthcare ecosystems introduces additional challenges related to cybersecurity, interoperability, and system reliability (12, 13). These challenges may erode user trust, hinder technology adoption, and compromise the reliability of digital healthcare services, making secure, controlled access to sensitive medical information a fundamental requirement for trustworthy digital healthcare systems (14, 15). Consequently, developing secure, privacy-preserving communication frameworks has become essential to ensuring the trustworthy, ethical, and sustainable use of digital health technologies.

Motivated by these challenges, this study proposes a privacy-preserving digital health communication and education framework that integrates social-network-based healthcare applications with a multi-cloud infrastructure, in which healthcare applications, data, and services are distributed across multiple independent cloud service providers rather than relying on a single cloud platform. This architecture is designed to enhance service availability, fault tolerance, reliability, and disaster recovery while reducing dependence on a single provider and supporting the secure storage, processing, and exchange of biomedical data across geographically distributed cloud environments. Building upon these capabilities, the proposed framework combines role-based privacy management with secure digital communication to enhance patient engagement, improve health literacy, facilitate effective collaboration among healthcare stakeholders, and promote the ethical and secure management of medical information.

The remainder of this paper is structured as follows. Section 2 presents related work in digital health communication and social healthcare systems. Section 3 introduces the proposed health communication and education framework. Section 4 discusses privacy-related challenges in digital health environments. Section 5 presents security considerations and mitigation strategies. Finally, Section 6 concludes the paper and outlines future research directions.

## 2 Related work

Recent advancements in digital health technologies have highlighted the growing importance of health communication, health education, and patient engagement through mobile and social-network-based platforms. Health literacy, defined by the World Health Organization as the cognitive and social skills that determine individuals’ motivation and ability to access, understand, and use health information to promote and maintain good health (16), is central to this shift. Following Nutbeam’s widely used typology, health literacy operates at three levels: functional health literacy, the basic reading, writing, and numeracy skills needed to understand health information; interactive health literacy, the ability to extract meaning from different forms of communication and apply it to changing circumstances; and critical health literacy, the ability to critically analyze health information and use it to exert greater control over health decisions (17). This distinction is significant because digital platforms do not support all three levels equally, and the framework proposed in this paper is designed with this distinction in mind. Several studies have explored the role of social networking systems in facilitating information exchange and improving communication between healthcare providers and patients. Prior research on social network analysis among healthcare providers has shown that structured provider-to-provider networks strengthen collaboration and improve coordination in clinical and community settings (18). Complementing this provider-side evidence, empirical research on community social capital has found that stronger community-level social ties are associated with improved access to healthcare (19).

From a public health perspective, empirical evidence supports the role of social and digital communication platforms in shaping health literacy outcomes. However, the strength and nature of this evidence varies by context and population. A systematic review of 98 empirical studies on social media use for health communication found that such platforms are widely used across patient, public, and professional populations, while also identifying significant limitations in the reliability and quality control of the health information shared on them (20). In the specific context of online health communities, a cross-sectional survey of 560 participants in Chinese online health communities found that patients’ eHealth literacy was positively associated with physician-patient communication and health information-seeking behavior, and, through these factors, with patient treatment adherence (21). Complementing this quantitative evidence, a case study of peer-to-peer health communication across clinical trial support networks, social media channels, and personal health blogs found that the benefits of peer information exchange are highly dependent on users’ existing health literacy. Users with lower baseline health literacy were less able to critically evaluate peer-generated health content, meaning that unstructured platforms can widen rather than close literacy gaps if not deliberately designed to support all three literacy levels (22). At the network-structure level, an empirical social network analysis of peer support connections in an online health community found that peer connections form more readily and become more interactive between users who share the same structural role, such as patient-to-patient or caregiver-to-caregiver, suggesting that role-aware platform design better supports meaningful peer communication than generic open social networking (23).

In addition, mobile health and wearable technologies have expanded the scope of continuous health monitoring and patient engagement, enabling real-time data collection and communication between patients and healthcare providers to support preventive care and early intervention (24). However, integrating such technologies into healthcare systems poses challenges related to data privacy, interoperability, and secure communication, which are examined further in this study.

Beyond these general findings on health communication and literacy, several existing technical frameworks have specifically addressed privacy and security in cloud-based and socially integrated healthcare systems, though each with a narrower scope than the framework proposed here. A framework for socially integrated mobile healthcare applications operating in a multi-cloud environment introduced customized, role-based privacy levels for participants and analyzed cybersecurity risks associated with combining social media features with mobile healthcare data, but did not incorporate a structured health education component or address health literacy outcomes (25). A multi-authority attribute-based access control scheme for cloud-based healthcare data sharing achieved fine-grained, cryptographically enforced access policies and demonstrated strong performance in access control efficiency and scalability, but focused narrowly on secure data-sharing mechanics rather than stakeholder communication, education, or emergency response workflows (26). Similarly, an attribute-based encryption scheme for personal health records in cloud computing introduced patient-centric, multi-authority access control with support for emergency break-glass access, offering strong cryptographic guarantees but, again, without addressing health communication or literacy as a system objective (27). Table 1 compares these existing systems with the framework proposed in this study across four dimensions: underlying architecture, privacy mechanism, health communication and education functionality, and stakeholder scope.

**Table 1:** Comparison of the proposed framework with existing systems.

Aspect	Al-Muhtadi et al. (25)	Gupta et al. (26)	Li et al. (27)	Proposed framework
Architecture	Multi-cloud, social-network-integrated mobile healthcare	Multi-authority cloud storage	Multi-authority cloud storage with emergency access	Multi-cloud, role-structured social communication
Privacy mechanism	Role-based privacy levels, communication minimization	Attribute-based encryption, policy privacy	Attribute-based encryption, security domains	Role-based access control, consent management, encryption, anonymization, audit trails, compliance
Health communication and education	Limited to privacy-aware social interaction	Not addressed	Not addressed	Explicit mechanisms for functional, interactive, and critical health literacy
Stakeholder scope	Patients, general social media users	Data owners, institutions	Patients, data owners, emergency responders	Patients, doctors, pharmacists, family members, paramedical staff, emergency services, medical students, administrators

Further, this comparison indicates that existing systems tend to specialize in either cryptographic access control for secure data sharing or privacy-aware social interaction. Still, none jointly address structured health communication, health literacy, and multi-stakeholder emergency response within a unified, role-based multi-cloud architecture. This is the specific gap the proposed framework addresses.

Overall, these findings collectively motivate and complicate the design of the proposed framework. On one hand, they confirm that digital and social platforms can meaningfully support health communication and health literacy. On the other hand, they show that these benefits are conditional. They depend on users’ baseline eHealth literacy (21), on whether platforms are designed to support critical evaluation of information rather than passive consumption (22), and on whether peer connections are structured around meaningful roles rather than left unstructured (23). This is a key gap that the proposed framework directly addresses. Rather than functioning as a generic social network, it embeds structured, role-based communication, since unstructured peer networks have been empirically shown to produce uneven health literacy benefits.

## 3 Digital health communication and education framework

The proposed framework introduces a privacy-preserving digital health communication and education system designed to enhance patient engagement, improve health literacy, and support timely healthcare decision-making through structured interaction among patients, healthcare providers, and family members. Unlike traditional healthcare information systems that focus primarily on clinical data management, this framework emphasizes health communication, patient education, and continuous health awareness through social-network-enabled healthcare environments (28–30). The system is designed to support both routine healthcare communication and emergency response scenarios, where timely information exchange among stakeholders is critical to patient outcomes. The framework integrates mobile health applications, social communication features, and multi-cloud infrastructure to ensure the continuous availability, scalability, and resilience of healthcare services. Within this model, patients actively participate in digital health communities where they can receive health-related guidance, share symptoms, and engage in structured communication with healthcare professionals. This interaction is expected to support informal health education and awareness, potentially enabling patients to better understand health-related information and support self-care practices. Healthcare providers, in turn, can monitor patient status through connected devices and communication channels, enabling early detection of health risks and timely intervention. Continuous physiological monitoring in this context builds on established principles of wireless sensor networks for low-power, distributed data collection (24), which underpin the connected devices used for patient monitoring.

The framework defines multiple stakeholders, including patients, healthcare professionals, family members, paramedical staff, emergency responders, medical students, and system administrators (Figure 1). Each stakeholder plays a specific role in ensuring effective communication flow and coordinated healthcare delivery. To maintain ethical data handling and ensure privacy protection, the system implements role-based access control, with access to medical information and communication features determined by predefined user roles and responsibilities. In emergency scenarios, the system enables automated escalation of alerts from patients’ monitoring devices to family members, primary physicians, and emergency medical services. If immediate response is not available, the system dynamically redirects alerts to alternative healthcare providers, ensuring continuity of care. Additionally, integration with location-based services supports ambulance dispatch and hospital coordination (31, 32).

**Figure 1:**
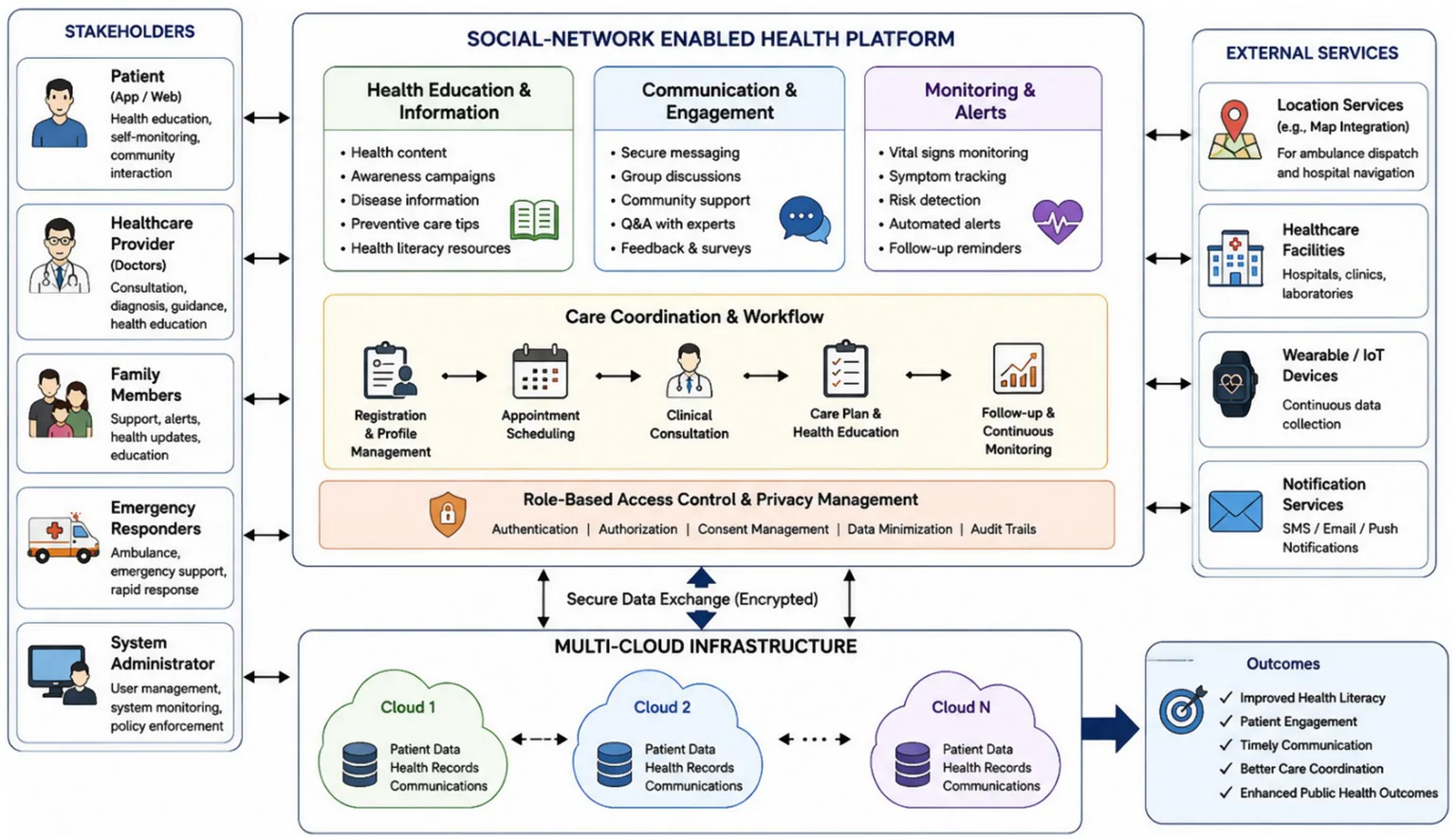
Overview of the healthcare system.

### 3.1 Health literacy mechanisms

The proposed framework addresses each of Nutbeam’s three health literacy dimensions (17) through distinct, identifiable mechanisms rather than treating health literacy as a general by-product of communication. Functional health literacy is supported through curated, plain-language health education content delivered directly through role-appropriate channels, providing patients with a baseline vocabulary and understanding of their condition. Interactive health literacy is supported through structured doctor-to-patient and pharmacist-to-patient communication channels that allow patients to ask clarifying questions and receive contextualized guidance rather than static, one-way information delivery. Critical health literacy is supported through community-based health discussion features and access to comparative, professionally reviewed educational content, enabling patients to evaluate the quality and relevance of health information rather than passively receive it. This directly addresses the risk that unstructured peer platforms can widen literacy gaps for patients with lower baseline literacy (22). While empirical validation of these mechanisms is outside the scope of this conceptual study, their effectiveness could be assessed in future implementations using validated instruments such as the Health Literacy Questionnaire or the eHealth Literacy Scale (21), alongside behavioral proxies such as patient engagement frequency, appointment adherence, and self-reported understanding of care instructions before and after framework use.

### 3.2 Privacy-preserving mechanisms

Privacy protection in the proposed framework operates through six coordinated mechanisms applied jointly across the three data sensitivity layers defined by the framework: health education data, health communication data, and sensitive clinical data. Role-based access control restricts data visibility and communication permissions according to stakeholder role, as summarized in Table 2, so that family members can view educational notifications and care-coordination updates while being structurally denied access to clinical decision data, and medical students can access anonymized educational cases without access to identifiable patient decision-making data. User consent management governs whether and how a given user’s data may be accessed, shared, or used, giving patients explicit control over the disclosure of their information. Data encryption protects information both at rest and in transit, applied with increasing strictness as data sensitivity increases. Data anonymization and pseudonymization limit the exposure of identifiable information for non-clinical or educational uses, such as when clinical cases are prepared for medical student training. Audit trails and activity logging record access events across the system to support accountability and post-hoc review. Privacy compliance mechanisms align data handling practices with applicable regulatory requirements, including data residency and cross-border data protection obligations.

**Table 2:** Role-based health communication and access in digital health education framework.

Role	Can do (health communication and education functions)	Cannot do (restricted actions)	Public health purpose
System Administrator	Manage system access, ensure secure health communication flow	Access clinical decision-making or modify medical prescriptions	Ensures a safe and ethical digital health environment
Doctor	Provide diagnosis guidance, health education, and care instructions	Unauthorized modification of system governance or patient privacy settings	Supports clinical communication and patient education
Pharmacist	Provide medication education and usage guidance	Change medical diagnoses or treatment plans	Improves medication literacy and safe use awareness
Patient	Access health education content, communicate symptoms, join health communities, and receive alerts.	Self-prescribe or alter clinical decisions	Enhances health literacy and self-care awareness
Family Members	Receive updates, support patient care coordination, and access educational notifications.	Access full clinical decision authority	Supports caregiving awareness and patient support
Paramedical Staff	Support patient monitoring and data collection	Make independent medical decisions	Improves care coordination and response efficiency
Medical Student	Access anonymized educational cases and learning content	Access identifiable patient decision-making data	Enhances medical education and training
Emergency Services	Receive alerts and coordinate emergency response	Modify patient medical records	Improves emergency response and health system coordination

These six mechanisms are applied with the degree of protection scaled to data sensitivity. Health education data, carrying low re-identification risk, is subject to minimal access restrictions to support broad reach and open availability. Health communication data is subject to role-based visibility rules that restrict community discussion access to relevant patient groups and limit family member access to coordination-relevant updates rather than full communication logs. Sensitive clinical data receives the strictest protection, combining encryption, anonymization for non-clinical uses, and audit logging to prevent misuse or unauthorized disclosure. This layered approach is consistent with prior mobile healthcare security research, which shows that trust-based, role-differentiated communication schemes reduce the risk of unauthorized data access in mobile health networks.

### 3.3 Multi-cloud architecture and comparative advantage

A key feature of the proposed framework is its secure multi-cloud architecture, which distributes storage across multiple independent cloud providers to ensure redundancy, availability, and fault tolerance. Medical records, communication logs, and monitoring data are distributed across cloud environments to enhance system reliability and reduce service disruption risks. This distributed storage also underpins the six privacy mechanisms described above by ensuring they remain operational even if a single provider experiences disruption or compromise.

Compared to single-cloud or on-premise healthcare IT deployments, this architecture offers three specific advantages. First, fault isolation ensures that a service disruption or breach at one cloud provider does not compromise data or service availability at another, unlike single-cloud deployments, where a provider-level outage can cause system-wide downtime. Second, regulatory flexibility allows healthcare organizations to align storage location with data residency regulations, for example, keeping clinical data within a required jurisdiction while distributing lower-sensitivity educational content more broadly, which is difficult to achieve with a single-provider architecture. Third, avoiding vendor lock-in allows healthcare organizations to renegotiate, migrate, or diversify cloud services without a full system redesign, thereby reducing long-term dependency risk. These advantages come with trade-offs, including increased architectural complexity and the need for cross-provider identity and access management, which the role-based governance model described above is designed to address.

Overall, the proposed framework aims to bridge the gap between digital health communication and health education by providing a structured environment designed to support patient engagement, potentially strengthen health literacy, and facilitate healthcare responsiveness while maintaining privacy and security controls. Table 2 summarizes the role-based functions of health communication and education.

### 3.4 Illustrative use case scenarios

To ground the framework’s abstract components in concrete operational settings, this section presents three representative use case scenarios.

#### 3.4.1 Scenario 1: Diabetes patient monitoring and education

A patient with type 2 diabetes uses the platform to log daily glucose readings via a connected monitoring device. The system routes this clinical health data directly to the patient’s endocrinologist, while separately surfacing relevant health education data, such as dietary guidance and medication adherence tips, through the patient’s education feed. When a glucose reading falls outside a clinically defined safe range, the system triggers a health communication alert to both the patient’s physician and a designated family member, consistent with the role-based permissions defined in Table 2. Over time, the patient’s interactions with educational content triggered by specific clinical events may support functional and interactive health literacy by connecting those events to actionable, contextualized information rather than generic content delivery.

#### 3.4.2 Scenario 2: Emergency response for an older adult

An older adult with a wearable fall-detection device experiences a fall at home. The device triggers an automated emergency alert, which the system escalates according to a defined hierarchy, first to the primary physician, then to a family member, and simultaneously to emergency medical services, along with location data to support ambulance dispatch. If the primary physician does not acknowledge the alert within a defined time window, the system dynamically redirects the escalation to an alternate on-call provider, ensuring continuity of response. This scenario illustrates the framework’s proposed multi-stakeholder escalation logic under real-time constraints and provides a theoretical rationale for considering fault-tolerant, low-latency multi-cloud infrastructure as a potentially appropriate architectural choice rather than a generic design option.

#### 3.4.3 Scenario 3: Post-discharge care coordination

Following hospital discharge, a patient transitions from clinician-managed inpatient care to home-based recovery. The discharging physician uploads structured care instructions as clinical health data, accessible to the patient’s primary care provider and pharmacist. At the same time, simplified recovery guidance is made available to patients and family caregivers as health education data. The pharmacist uses the platform to provide medication usage guidance, consistent with their defined role in Table 2, and family members receive care-coordination updates without access to full clinical records. This scenario illustrates how the proposed three-tier data model is designed to support coordinated, role-appropriate information flow across a care transition, a point in the care pathway where communication breakdowns are well documented as a source of adverse outcomes.

### 3.5 Proposed hypotheses and theoretical propositions

The proposed framework establishes theoretical relationships among health communication, health literacy, privacy protection, patient engagement, and multi-cloud infrastructure. Because the framework is conceptual and has not yet been empirically implemented or validated, the relationships presented below are formulated as testable hypotheses rather than established effects. These hypotheses provide a basis for future empirical studies evaluating the proposed framework in real-world digital healthcare environments.

*H1:* Privacy-preserving mechanisms are hypothesized to positively influence users’ trust in digital health communication and education systems.

The framework incorporates role-based access control and privacy-preserving mechanisms to regulate access to sensitive biomedical information according to stakeholder roles. By providing controlled access to health information and limiting unauthorized disclosure, these mechanisms are expected to strengthen users’ confidence in the privacy and responsible management of their health data.

*H2:* Structured digital health communication is hypothesized to support patient engagement and health literacy.

The framework integrates health education mechanisms with digital communication pathways that connect patients with healthcare professionals, caregivers, and other relevant stakeholders. By providing role-appropriate access to health information and connecting educational content with healthcare-related communication, the framework is designed to support active patient participation and the understanding and use of health information.

*H3:* A multi-cloud architecture is hypothesized to support the availability, reliability, scalability, and resilience of digital healthcare services.

The proposed framework distributes healthcare data and services across multiple independent cloud providers rather than relying exclusively on a single provider. This architecture is designed to reduce dependence on a single provider and potential single points of failure while supporting continuity of healthcare communication and information services. The extent to which these expected advantages are achieved requires empirical evaluation through technical benchmarking and real-world deployment.

*H4:* The integration of privacy-preserving mechanisms, structured health communication, and multi-cloud infrastructure is hypothesized to support secure and responsible biomedical data sharing among healthcare stakeholders.

The framework combines privacy controls, role-based access, health communication pathways, and distributed cloud infrastructure to establish a coordinated environment for exchanging biomedical and health education information. This integrated approach may support the balance between information accessibility required for healthcare coordination and the confidentiality and privacy requirements of patients and other stakeholders.

Collectively, these hypotheses represent the principal theoretical relationships underlying the proposed framework. H1 addresses the expected relationship between privacy protection and user trust, H2 addresses the relationship between structured digital health communication and patient engagement and health literacy, H3 addresses the expected contribution of multi-cloud architecture to system-level resilience, and H4 addresses the combined role of these mechanisms in secure and responsible biomedical data sharing. These relationships are proposed for future empirical testing and should not be interpreted as validated outcomes of the present conceptual study.

## 4 Privacy and health communication challenges

The increasing adoption of digital health communication and education systems has introduced new challenges related to data sensitivity, privacy perception, and trust in healthcare interactions. Unlike traditional healthcare systems that focus primarily on clinical data processing, digital health communication platforms involve multiple layers of information exchange, including educational content, interpersonal communication, and clinical data sharing. These challenges are not unique to healthcare. Empirical privacy research on general-purpose online social networks has shown that unauthorized data access and privacy leakage are systemic risks in any platform that supports open information exchange between users (33). Analysis of privacy practices on major social networking platforms has further shown that users are frequently unaware of how their shared information propagates beyond their intended audience, and that platform-level privacy controls are often insufficient to prevent unintended disclosure (34). These findings are directly relevant to digital health communication platforms, which combine the open, network-based interaction patterns of general social platforms with substantially more sensitive data, thereby raising the privacy stakes considerably above those in non-health social networking contexts.

As illustrated in Figure 2, health-related data in digital health environments is classified into three layers based on sensitivity and intended use: health education data, health communication data, and sensitive clinical data. Health education data represents the lowest sensitivity level and includes health education content, health tips and awareness material, preventive messages, community resources, and public health campaigns. Its purpose is to improve health literacy, awareness, and preventive behaviors. Health communication data has a medium sensitivity level and includes doctor-to-patient messages, family updates and discussions, alert notifications, appointment reminders, and community discussions. Its purpose is to enable effective health communication and stakeholder engagement. Sensitive clinical data represents the highest level of sensitivity and includes medical records, diagnostic information, laboratory reports and imaging, treatment plans, and insurance and billing details. Its purpose is to support clinical care and treatment decisions.

**Figure 2:**
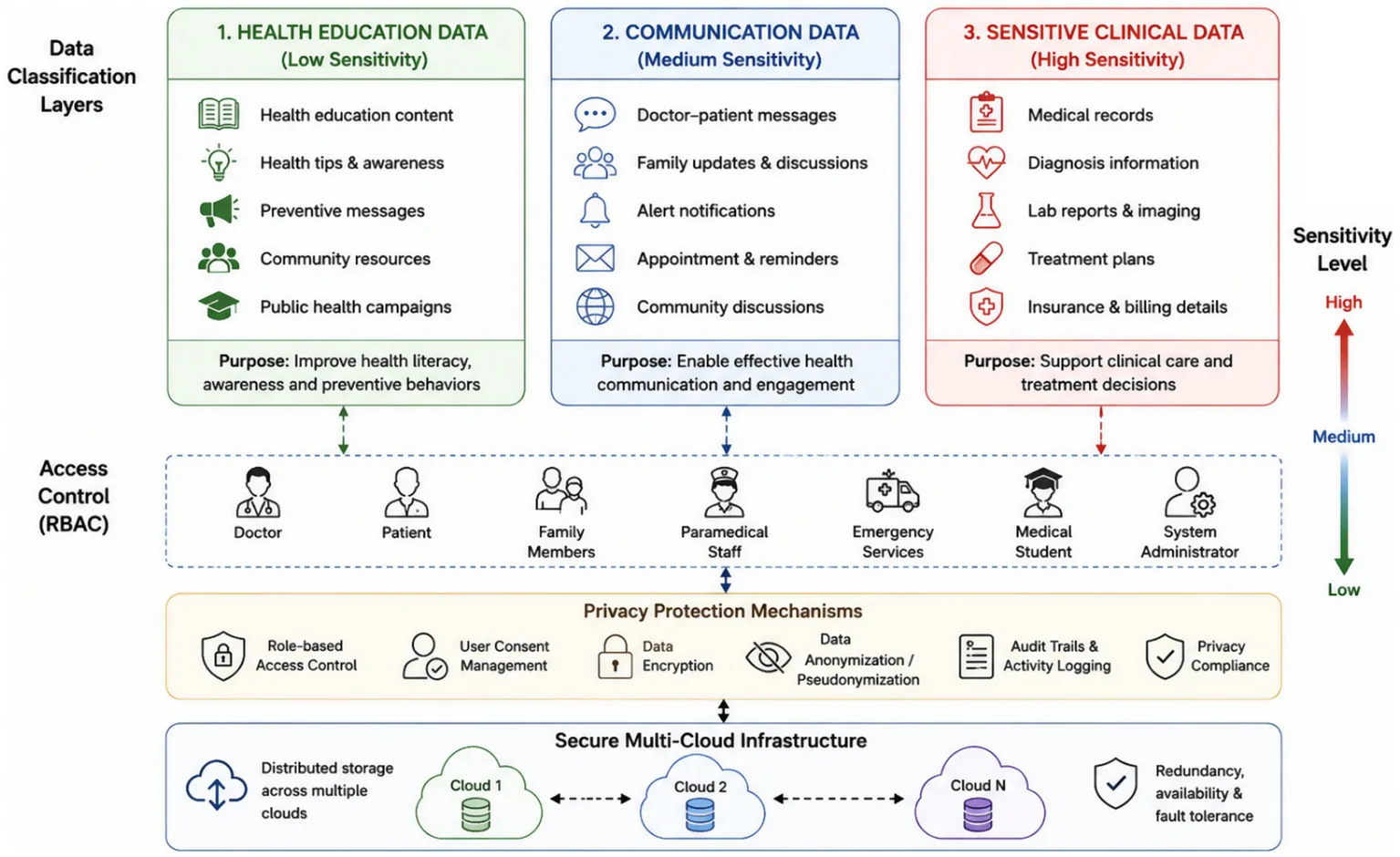
Health data classification in digital health communication and education systems.

Access to these three data layers is governed by a shared role-based access control structure spanning doctors, patients, family members, paramedical staff, emergency services, medical students, and system administrators, so that each stakeholder’s visibility into a given data layer corresponds to their functional role rather than being granted uniformly across the system. This access-control structure is supported by six coordinated privacy protection mechanisms: role-based access control, which restricts data visibility according to stakeholder role; user consent management, which governs whether and how a user’s data may be accessed or shared; data encryption, which protects information both at rest and in transit; data anonymization and pseudonymization, which limit the exposure of identifiable information for non-clinical or educational uses; audit trails and activity logging, which record access events to support accountability; and privacy compliance mechanisms, which align data handling with applicable regulatory requirements. These mechanisms operate jointly across all three data layers, with the degree of protection applied scaled to the sensitivity of the corresponding data.

Underpinning this structure is a secure multi-cloud infrastructure that distributes storage across multiple independent cloud providers. This distribution provides redundancy, availability, and fault tolerance, ensuring that health communication and education services, as well as the privacy protection mechanisms that govern them, remain operational even if a single cloud provider experiences disruption or compromise.

From a public health perspective, this layered data classification highlights that privacy is not only a technical requirement but also a communication and trust-building factor in digital health systems. Users’ willingness to engage with health platforms is strongly influenced by their perception of how their information is used, shared, and protected, a dynamic already documented in general social network contexts (34) and amplified in healthcare settings, where the consequences of unauthorized disclosure extend beyond reputational harm to potential discrimination, insurance implications, and loss of trust in care providers. Improper handling of any data layer may reduce trust, limit participation, and negatively affect health education outcomes. Secure transfer mechanisms that preserve data integrity as information moves between monitoring devices, communication channels, and decision-makers are therefore essential, not only to protect confidentiality but also to ensure that the health information stakeholders act on has not been tampered with in transit (28–30). Ensuring appropriate handling of data at different sensitivity levels is therefore essential for maintaining effective digital health communication environments. The proposed framework emphasizes responsible information sharing, user awareness, and trust-centered communication practices, operationalized through the access control, privacy protection, and multi-cloud mechanisms described above, to support sustainable engagement in digital health education systems.

## 5 Risks to health communication and education systems

Digital health communication and education systems play an essential role in improving health literacy, enhancing patient engagement, and supporting coordinated healthcare delivery. However, the effectiveness of these systems can be influenced by various risks related to communication integrity, trust, information reliability, and user behavior. In social-network-based healthcare environments, users interact continuously through messages, alerts, and health education content. While this interaction improves accessibility and engagement, it also introduces risks that may affect the quality and reliability of health communication. These risks include identity misuse, the dissemination of misinformation, unauthorized access to personal health-related information, and reduced credibility of health content sources.

As summarized in Table 3, these risks can be categorized based on their impact on health communication and education outcomes, and each category corresponds to a distinct body of technical work on detection and mitigation. For instance, identity risks, such as impersonation or fake profiles, can spread misleading health information and reduce trust in digital health education platforms. Detecting such risks typically relies on structural analysis of user interaction patterns, for example, identifying accounts whose connection patterns deviate from those of genuine patient or caregiver users, a detection approach grounded in established social network analysis methods. This structural approach complements role-based access controls, since verified role assignment, such as confirming a user is a licensed pharmacist before granting pharmacist-level permissions, directly reduces the surface for identity-based misuse. Communication risks, such as message distortion or the spread of misinformation, can negatively affect patient understanding and decision-making. Detecting these risks at the network level draws on network forensics techniques that analyze network traffic and content flows to trace the origin and propagation of manipulated or distorted communications. Related detection methods, originally developed for identifying spam and unsolicited content in general network traffic, are also applicable to filtering low-credibility or manipulated health content before it reaches patients and community members.

**Table 3:** Risks to digital health communication and health education systems.

Category	Threat type	Impact on health communication and education
Identity risks	User impersonation, fake profiles	Reduced trust and misinformation in health education platforms
Communication risks	Message distortion, misinformation spread	Incorrect health understanding and poor decision-making
Privacy risks	Unauthorized access to personal health data	Reduced user engagement and trust
Behavioral risks	Phishing, social engineering	Lower participation in digital health systems
System risks	Service disruption, overload	Interrupted access to health education and alerts
Trust risks	Low credibility of information sources	Reduced adoption of health communication platforms

Privacy risks, including unauthorized access to personal health data, may discourage users from actively participating in digital health systems, particularly when there is uncertainty about how personal health information is used or shared, a concern consistent with prior privacy-leakage findings in online social networks. Mitigating these risks depends on robust encryption and data-protection mechanisms. Cryptographic sequence-generation techniques, as used in secure enciphering schemes, provide one technical basis for protecting clinical health data at rest and in transit. Behavioral risks, such as phishing or social engineering attempts, may further erode user confidence and engagement on health communication platforms. These attacks often exploit trust relationships between stakeholders, for example, an attacker impersonating a family member or care coordinator, making them harder to detect through content analysis alone and reinforcing the need for identity verification and detection methods. System risks, such as service disruptions or overload, may limit access to timely health education content and emergency alerts, affecting the continuity of care and communication. A multi-cloud architecture can directly mitigate this risk category by isolating faults across independent providers, reducing the likelihood that a single point of failure disrupts time-critical services such as emergency alert escalation. Trust risks arising from the low credibility of information sources can reduce the adoption of health communication platforms. Platforms that support critical health literacy, enabling users to evaluate the credibility of health information rather than passively accept it, are structurally better positioned to withstand trust erosion than platforms that deliver content without supporting critical evaluation (22).

Overall, these six risk categories reinforce the idea that privacy, access control, and multi-cloud mechanisms are not independent design choices but interlocking responses to a specific, identifiable set of threats to health communication and education outcomes.

## 6 Conclusion

This study proposed a privacy-preserving digital health communication and education framework that integrates social-network-enabled healthcare applications with a multi-cloud infrastructure to support the secure, ethical, and efficient management of biomedical information. The framework makes four main contributions. First, it unifies health communication, health education, and role-based access control within a single architecture, enabling secure interaction among patients, healthcare professionals, caregivers, and emergency responders. Second, by embedding privacy-preserving mechanisms directly into communication pathways, the framework is designed to support patient engagement, health literacy, and trust in digital healthcare services. Third, the study proposes that a multi-cloud architecture may support availability, reliability, scalability, and resilience by distributing healthcare services and data across multiple independent cloud providers, thereby reducing dependence on a single provider and potential single points of failure. Fourth, the proposed governance and access control mechanisms establish a foundation for ethical data sharing and illustrate how privacy and trust requirements may be operationalized in digital health environments rather than treated solely as abstract design goals.

Despite these contributions, the study has several limitations that should be acknowledged. The framework is conceptual in nature and has not yet been implemented or validated in a real-world healthcare environment, so its performance under operational conditions remains unverified. The evaluation relies on qualitative analysis of healthcare communication workflows and privacy requirements rather than quantitative experiments, benchmarking, or user-based assessments, which limits claims about efficiency, usability, or measurable security gains. Additionally, the framework does not yet account for context-specific regulatory variation across jurisdictions, such as HIPAA and GDPR, nor does it address integration challenges with legacy healthcare IT systems, which are common barriers to real-world adoption. These limitations mean the framework should currently be viewed as a design and governance model rather than a validated system.

From a policy perspective, the proposed framework offers concrete guidance for healthcare organizations, policymakers, and digital health developers. It highlights the need for privacy-by-design principles in the development of social-network-enabled healthcare tools, rather than treating privacy as a compliance afterthought. It also underscores the value of role-based, multi-stakeholder access-control models in balancing data accessibility for care delivery with confidentiality obligations. For policymakers, the framework illustrates how governance structures may be designed to support ethical data sharing across cloud providers while remaining adaptable to evolving healthcare privacy regulations. For healthcare organizations, it provides a reference model for evaluating vendor and cloud-provider arrangements against resilience, availability, and data-sovereignty requirements.

Building on these findings, future research should pursue several specific directions. First, the framework requires empirical validation through pilot implementation in real-world healthcare settings, using real or simulated healthcare datasets and structured user studies with patients, clinicians, and caregivers to assess usability and communication effectiveness. Second, quantitative performance evaluation is needed, including benchmarking of latency, scalability, and fault tolerance across multi-cloud configurations under realistic workloads. Third, future work should integrate machine learning and deep learning techniques to enable intelligent privacy management, such as anomaly detection in access patterns, personalized health communication, predictive analytics for patient risk stratification, and adaptive health education content. Fourth, further investigation is needed into interoperability with existing electronic health record systems and healthcare standards such as HL7 FHIR, formal regulatory compliance auditing, and secure multi-cloud deployment strategies, including key management and cross-provider trust mechanisms. Together, these directions would strengthen the framework’s practical applicability and support its transition from a conceptual model to a deployable system for large-scale healthcare ecosystems (35, 36).

## Data Availability

The original contributions presented in the study are included in the article/supplementary material, further inquiries can be directed to the corresponding author.
